# Validation of a Patient‐Created, Patient‐Reported Experience Measure for Inflammatory Bowel Disease in the United Kingdom

**DOI:** 10.1111/hex.70814

**Published:** 2026-09-09

**Authors:** Zoe Guy, Daniel Hind, Nikki Totton, Elena Sheldon, Naseeb Ezaydi, Matt Bursnall, Alan Lobo

**Affiliations:** ^1^ Clinical Trials Research Unit The University of Sheffield Sheffield UK; ^2^ Leeds Institute of Health Sciences Leeds UK; ^3^ Sheffield Health and Social Care NHS Foundation Trust Sheffield UK; ^4^ Sheffield Inflammatory Bowel Disease Centre Sheffield Teaching Hospitals NHS Foundation Trust Sheffield UK

**Keywords:** IBD, patient experience, patient‐led development, validation

## Abstract

**Introduction:**

Inflammatory bowel disease (IBD) significantly impacts quality of life and has high healthcare contact. Ensuring that patients are experiencing the best care is therefore essential and has been the focus of many quality improvement initiatives. To support this, in collaboration with patients, we developed a patient‐reported experience measure for IBD (IBD‐PREM) for use in a UK context. This article refers to the validation of this patient‐developed measure to ensure it is valid and reliable.

**Methods:**

Using data from the AWARE‐IBD study, 287 participants provided data between November 2021 and November 2024. The IBD‐PREM as well as the IBD‐Control questionnaire was collected at 3‐month intervals as well as 2 weeks post baseline. Validation was completed through testing the following aspects: acceptability (response rate and missing data rates), reliability (internal consistency and test‐retest reliability), validity (construct and structural validity using factor analysis) and responsiveness (ceiling/floor effects and smallest detectable difference).

**Results:**

High response rates and low missing data demonstrated acceptability of the IBD‐PREM. There was also good test‐retest reliability (ICC = 0.88). Low ceiling and floor effects demonstrate suitable responsiveness as did the ability to differentiate between patients with active and inactive disease (effect size = 0.62). The confirmatory factor analysis suggested the current domain structure (three domains) is not optimum and factor loadings indicated some potentially redundant items, which is supported by the high internal consistency (*α* = 0.97).

**Conclusions:**

The patient‐developed IBD‐PREM is a tool that can be used as a method for assessing the healthcare experiences of IBD patients in the United Kingdom. Superior psychometric properties were found compared to measures developed through traditional researcher‐led approaches suggesting the benefit of involving patients early in the methodological process. Further refinement in terms of item reduction and domain restructure should be implemented to optimise the PREM as well as external validity to other contexts.

**Patient or Public Contribution:**

Patients and members of the public were engaged from an early stage in the development of the PREM, contributing directly to the design and content of the IBD‐PREM. This article goes on to validate this patient‐created measure with input from a patient representative.

## 1 Introduction

Inflammatory bowel disease (IBD), including Crohn's disease and ulcerative colitis, is characterized by chronic inflammation of the gastrointestinal tract. IBD significantly impacts quality of life and psychosocial functioning due to its unpredictable trajectory of active disease and remission [1].

Patient and healthcare provider views often differ regarding care priorities and quality, with patients often rating their care quality as poor [2]. Many IBD quality improvement initiatives recognize the importance of learning from patients [3] and patient‐reported measures are increasingly used as quality indicators [4], including in IBD [5]. However, not all self‐complete survey instruments are considered useful or effective [6]. Patient satisfaction measures, which assess whether care met expectations, can be biased by past experiences [6, 7], lack sensitivity to distinguish good versus bad care, and tend to overrate satisfaction [8]. High reported satisfaction may therefore not equate to a positive care experience [7]. Patient‐Reported Experience Measures (PREMs) are reported to more accurately represent healthcare quality by capturing what actually happened during care, how, and how often [9, 10, 11]. PREMs therefore provide actionable targets for improvement based on what matters most to patients [4, 6, 9, 12].

In previous work, we critically reviewed existing instruments for IBD specifically considering an aspect of patient experience [13]. Within this work, we identified 20 instruments that covered five main topics: patient satisfaction [5], knowledge [14], concerns [15], self‐efficacy [16] and perceived quality [17]. However, no instrument was found to include all aspects of experience, based on the patient experience framework. The conclusions of this work was that ‘patient experience of IBD management is multifaceted and incompletely represented by individual survey instruments’ [13].

In an attempt to address this gap in ability to directly collect experience information, we created an IBD‐PREM which integrated multiple conceptual frameworks allowing it to capture both service interactions and behavioural determinants [18]. This PREM was co‐produced with IBD service users to ensure its appropriateness. Experience measures have been co‐produced with success in other conditions such as: Intellectual Disability [19], Hearing Loss [20], Older adults in Emergency Departments [21], and Diabetes [22]. The development, as described elsewhere [18], consisted of taking a long list of survey instrument items and completed four workshops in 2021 with eight service users to reduce, amend and adjust the PREM.

The final 38‐item IBD‐PREM consisted of three domains, as decided by the IBD service users,—‘the care team’, ‘what matters to me’, and ‘living with Crohn's or Colitis’. This measure is intended to be used as a quality indicator for a service either for comparison between services or to assess quality improvement initiatives. Within the initial work, the IBD‐PREM was mapped to 28 of 38 IBD UK standards [23], demonstrating how it can provide patient‐reported evidence of meeting quality standards for service evaluation. Additionally, the domains align with health system quality imperatives [24, 25].

In this paper, we aim to validate the IBD‐PREM by assessing its acceptability, reliability (internal consistency and test‐retest), validity (known groups and criterion validity), structural validity through factor analysis, responsiveness, and interpretability. The results were reviewed with a patient representative to contextualise the findings and identify further refinements to the instrument.

## 2 Materials and Methods

This validation work has been guided by the COSMIN definitions created for the measurement properties of patient‐reported outcome measures [26].

### 2.1 Participants

Data collected within the AWARE‐IBD study has been used to validate the PREM. The AWARE‐IBD study was a longitudinal, quality‐improvement study funded by The Health Foundation [27]. The study was completed in a single tertiary centre in the United Kingdom between October 2021 and November 2024. The purpose of the AWARE‐IBD study was to improve care for IBD patients by completing research led by what matters the most to patients. This is completed through the co‐production of this PREM which will collect data on aspects of importance to patients as well as considering them in the development of service change initiatives which can be tested in practice [28].

Inclusion criteria of participants in the AWARE‐IBD study, and therefore this validation dataset, were: 18 years or over, diagnosis of CD or UC based on conventional clinical, radiological or histological criteria (presumptive or confirmed) and ability to understand the study documentation.

### 2.2 Data Collection

Recruitment began in November 2021 and closed in May 2023. To allow at least 18 months of data for all participants, the final data collection took place in November 2024. In total, 287 participants were recruited to the AWARE‐IBD study, of which all data were used within this validation study. Data were collected directly from participants through a specifically designed web‐based application. Postal questionnaires were also accepted. Participants completed data at baseline, 2 weeks and then every 3 months post baseline until the end of the study. Table 1 shows the number of responses to each questionnaire per timepoint.

**Table 1:** Number and percentage of participants that responded and completed the IBD‐PREM, IBD‐Control and Anchor question per timepoint and the overall number of questionnaires.

Measure	Baseline	2 Week	3 Months	6 Months	9 Months	12 Months	15 Months	18 Months	21 Months	24 Months	Total
PREM responses	277 (97%)	259 (90%)	232 (81%)	193 (67%)	196 (68%)	196 (68%)	187 (65%)	145 (51%)	114 (40%)	95 (33%)	1894
Complete Care Team domain	256 (89%)	244 (85%)	213 (74%)	178 (62%)	178 (62%)	175 (61%)	174 (61%)	138 (48%)	100 (35%)	89 (31%)	1745
Complete What Matters to Me domain	221 (77%)	212 (74%)	187 (65%)	150 (52%)	160 (56%)	152 (53%)	150 (52%)	114 (40%)	89 (31%)	74 (26%)	1509
Complete Living with C&C domain	250 (87%)	236 (82%)	207 (72%)	171 (60%)	176 (61%)	169 (59%)	170 (59%)	129 (45%)	99 (34%)	86 (30%)	1693
Complete PREM	215 (75%)	204 (71%)	181 (63%)	148 (52%)	155 (54%)	146 (51%)	146 (51%)	112 (39%)	87 (30%)	73 (25%)	1467
IBD‐Control responses	271 (94%)		228 (79%)	189 (66%)	197 (69%)	192 (67%)	183 (64%)	145 (51%)	110 (38%)	92 (32%)	1607
Complete IBD‐Control‐8	235 (82%)		202 (70%)	168 (59%)	172 (60%)	162 (56%)	164 (57%)	129 (45%)	100 (35%)	85 (30%)	1417
Complete IBD‐Control VAS	266 (93%)		224 (78%)	187 (65%)	195 (68%)	188 (66%)	181 (63%)	143 (50%)	108 (38%)	92 (32%)	1584
Anchor responses		252 (88%)				192 (67%)					444
Complete Anchor		249 (87%)				191 (67%)					440

The AWARE‐IBD study received ethical approval from Wales REC 3 (ref 298930) to collect data for the purposes of both quality improvement projects and measure validation. Participants gave informed consent prior to providing any data. Participants were not compensated for taking part in the study.

### 2.3 Outcome Measures

Data collected at each timepoint consisted of both the IBD‐PREM and the IBD‐Control (see below for more details). At 2 weeks and 12 months, participants were also asked an experience anchor question.

### 2.4 IBD‐PREM

As defined in the original paper which outlined its creation [18], the IBD‐PREM is a 38‐item measure with two questions consisting of multiple parts (item 18 with four parts and item 21 with five parts), resulting in 45 questions overall. The presentation of the questionnaire was standard for all responses and timepoints, no difference in presentation or randomisation occurred. The measure includes three domains:

- The care team (item 1–5, 5 items)
- What matters to me (item 6–26, 21 items, 28 questions)
- Living with Crohn's or Colitis (item 27–38, 12 items).

Responses are measured on a Likert scale from 1 to 5 ranging from ‘not at all’ to ‘to a very large extent’. The full IBD‐PREM can be found in Appendix 1. Domain scores and a total score are calculated using the mean of all relevant items. Scores are between one and five with higher scores indicating a better experience. No missing data imputation was applied to the validation and therefore domain and total scores were only calculated for complete responses.

### 2.5 IBD‐Control Questionnaire

The IBD‐Control questionnaire is a 13‐item questionnaire and a 100‐point visual analogue scale aiming to measure disease control for IBD patients [29]. Individual items (apart from the VAS) are scored from zero to two. The total score (IBD‐Control‐8) is calculated by summing eight of the responses, resulting in a total score range from zero to 16, where better scores indicate better control. For consistency, no missing data imputation was applied to the IBD‐Control‐8 so the total score was calculated for complete responses only.

### 2.6 Experience Anchor Question

The experience anchor question asked, ‘since you entered the study, has there been any change in your overall experience of the service?’. This allowed response options of: ‘worse, about the same, or better’ with a follow‐on question to evaluate the magnitude of this difference (Likert scale from one to seven). This will be used to identify a stable subgroup of patients for the test‐retest reliability [30], as well as a minimal change subgroup for the minimum important difference (MID) calculation [31]. The format of the question considered important aspects such as reflecting on the patient's current state.

### 2.7 Analysis

#### 2.7.1 Patient Characteristics

Characteristics of the participants that provided data for the PREM validation are summarised using appropriate descriptive statistics.

#### 2.7.2 Acceptability

Acceptability is described using summaries of the submission duration (online responses only), response rate for the questionnaire, as well as completion rates for individual items and domains.

#### 2.7.3 Reliability

Reliability was assessed through two different mechanisms: internal consistency and test‐retest reliability. Internal consistency was assessed using Cronbach's alpha over the items within each of the domains as well as across all items. Test‐retest reliability took data from participants that report no change on the experience anchor question at 2‐weeks and calculated the intraclass correlation coefficient (ICC), as well as mean differences between baseline and 2‐weeks. Additionally, a Bland‐Altman plot was produced to visually represent any differences. Again, this was completed on the total score as well as each of the three domains individually.

#### 2.7.4 Validity

Validity was assessed using construct and criterion validity. Content validity was previously completed and reported during the creation of the IBD‐PREM [18]. Under construct validity, known groups validity was used with a singular hypothesis due to the limited literature available on patient experience and the available data. This hypothesis, that patients with active disease (as defined by having an IBD‐Control‐8 score of less than 13) will have a lower PREM score on the living with Crohn's or Colitis domain, was tested using an independent *t*‐test. Structural validity was measured using confirmatory factor analysis (CFA) assuming the three domains in the first instance. Standard model fit assessment was used [32] and non‐optimal fit of the three domains was investigated through exploratory factor analysis (EFA) to consider alternative solutions.

Criterion validity used a Pearson's correlation coefficient on the total PREM score and the total IBD‐Control‐8 score across all timepoints. Although this was not expected to be perfectly correlated, it is the most appropriate ‘gold standard’ available and therefore a moderate correlation was expected.

Cross‐cultural validity was not measured as this work was completed within the United Kingdom in English only and therefore further work to assess its validity beyond this context will need to be completed separately.

#### 2.7.5 Responsiveness

Responsiveness was assessed by calculating the proportion of ceiling (score = 5) and floor (score = 1) effects for the total score and each domain as well as the correlation between change scores for the PREM and the IBD‐Control (baseline to 12 months).

The smallest detectable difference was assessed using participants with PREM data at baseline and 2 weeks, a range for which we assumed there would be no difference in response. The smallest detectable difference was based on the standard error of measurement (SEM) [33, 34].

#### 2.7.6 Interpretability

Calculations of the MID used the anchor‐based method using a coded version of the experience anchor question (0 for no change, ±1 for a small negative/positive change, etc.). This was correlated with the PREM and the difference between baseline and 12 months for those that scored ‘a little bit better/worse’ was used to suggest a MID. The distribution‐based method was also used by calculating half of the standard deviation as an alternative.

#### 2.7.7 Thresholds

The threshold values deemed as acceptable for each of the tests within the analysis are outlined in Table A Appendix 2.

#### 2.7.8 Results

##### 2.7.8.1 Characteristics of the Participants

There were 287 participants who contributed data to the validation analysis. The median age of respondents was 52 years (range 18–89 years; IQR 38–63 years) and there was a greater proportion of female participants (62.7%). The majority of respondents self‐reported their ethnicity as White British (89.2%). Most participants' IBD subtype were either Crohn's Disease (49.1%) or Ulcerative Colitis (47.4%) and the median self‐reported time since diagnosis was 11 years (IQR 4–23 years) (Table 2).

**Table 2:** Baseline characteristics of participants recruited to complete the PREM.

Characteristic		Total *N* (%) *N* = *287*
Age (years)	*n*	287
	Mean (SD)	51.25 (15.98)
	Median (IQR)	52.00 (38.00, 63.00)
	Min, Max	18.00, 89.00
Sex	Female	180 (62.7%)
	Male	107 (37.3%)
Ethnicity	White British	256 (89.2%)
	Pakistani	5 (1.7%)
	Other	9 (3.1%)
	Not known	17 (5.9%)
Religion	Christian	154 (53.7%)
	No religion	102 (35.5%)
	Muslim	7 (2.4%)
	Other	5 (1.7%)
	Not known	19 (6.6%)
Index of Multiple Deprivation	*n*	287
	Mean (SD)	5.77 (3.02)
	Median (IQR)	6.00 (3.00, 8.00)
	Min, Max	1.00, 10.00
IBD Subtype	Crohn's Disease	141 (49.1%)
	Ulcerative Colitis	136 (47.4%)
	Inflammatory bowel disease unclassified/indeterminate colitis	9 (3.1%)
	Not known	1 (0.3%)
Time since diagnosis (years)	*n*	273
	Mean (SD)	15.09 (13.27)
	Median (IQR)	11.00 (4.00, 23.00)
	Min, Max	0, 59.00

##### 2.7.8.2 Acceptability

Summary statistics of the PREM items, domains and total score are presented in Table 3. The timepoints of particular interest for validation were baseline, 2 weeks and 12 months with respective response rates of 96.5%, 90.2% and 68.3% for the 287 participants who contributed. Of the 1894 responses received across all timepoints, 1733 (91.5%) were administered online and 161 (8.5%) by post. Median submission duration was 284 s (IQR 213–392 s) for online submissions. Table 3 shows that the majority of items had low rates of missing data; there were only six items with missing data greater than 5% and a maximum of 15.1% missing data for one item. Overall data completeness for the total PREM score was 77.5% (i.e., 22.5% missing at least one item), and rates of completeness for the domains were 92.1%, 79.7% and 89.4% for the ‘care team’, ‘what matters to me’ and ‘living with Crohn's or Colitis’ domains, respectively.

**Table 3:** Summary statistics of the PREM item, domain and total scores.

Variable	*n N* = 1894	Mean (SD)	Median (IQR)	Min, Max	Responses missing *N* (%)
Q1	1884	3.30 (1.06)	3.00 (3.00, 4.00)	1, 5	10 (< 1%)
Q2	1885	4.12 (0.93)	4.00 (4.00, 5.00)	1, 5	9 (< 1%)
Q3	1762	3.95 (1.02)	4.00 (3.00, 5.00)	1, 5	132 (7.0%)
Q4	1843	4.04 (0.91)	4.00 (4.00, 5.00)	1, 5	51 (2.7%)
Q5	1867	3.15 (1.36)	3.00 (2.00, 4.00)	1, 5	27 (1.4%)
Q6	1865	3.75 (0.99)	4.00 (3.00, 4.00)	1, 5	29 (1.5%)
Q7	1867	4.22 (0.86)	4.00 (4.00, 5.00)	1, 5	27 (1.4%)
Q8	1848	3.99 (0.98)	4.00 (3.00, 5.00)	1, 5	46 (2.4%)
Q9	1874	3.96 (1.03)	4.00 (3.00, 5.00)	1, 5	20 (1.1%)
Q10	1843	3.53 (1.08)	4.00 (3.00, 4.00)	1, 5	51 (2.7%)
Q11	1873	4.02 (1.01)	4.00 (3.00, 5.00)	1, 5	21 (1.1%)
Q12	1863	4.22 (0.86)	4.00 (4.00, 5.00)	1, 5	31 (1.6%)
Q13	1868	3.97 (1.00)	4.00 (3.00, 5.00)	1, 5	26 (1.4%)
Q14	1848	3.86 (1.06)	4.00 (3.00, 5.00)	1, 5	46 (2.4%)
Q15	1868	4.47 (0.71)	5.00 (4.00, 5.00)	1, 5	26 (1.4%)
Q16	1850	3.45 (1.21)	4.00 (3.00, 4.00)	1, 5	44 (2.4%)
Q17	1863	3.80 (1.15)	4.00 (3.00, 5.00)	1, 5	31 (1.6%)
Q18a	1768	3.70 (1.08)	4.00 (3.00, 4.00)	1, 5	126 (6.7%)
Q18b	1608	3.19 (1.20)	3.00 (3.00, 4.00)	1, 5	286 (15.1%)
Q18c	1836	3.35 (1.10)	3.00 (3.00, 4.00)	1, 5	58 (3.1%)
Q18d	1625	3.20 (1.14)	3.00 (3.00, 4.00)	1, 5	269 (14.2%)
Q19	1656	3.38 (1.19)	3.00 (3.00, 4.00)	1, 5	238 (12.6%)
Q20	1875	4.24 (0.83)	4.00 (4.00, 5.00)	1, 5	19 (1.0%)
Q21a	1867	4.29 (0.82)	4.00 (4.00, 5.00)	1, 5	27 (1.4%)
Q21b	1853	4.12 (0.96)	4.00 (4.00, 5.00)	1, 5	41 (2.2%)
Q21c	1850	4.17 (0.92)	4.00 (4.00, 5.00)	1, 5	44 (2.3%)
Q21d	1856	4.16 (0.99)	4.00 (4.00, 5.00)	1, 5	38 (2.0%)
Q21e	1855	4.11 (0.96)	4.00 (4.00, 5.00)	1, 5	39 (2.1%)
Q22	1803	3.37 (1.28)	4.00 (3.00, 4.00)	1, 5	91 (4.8%)
Q23	1845	3.25 (1.41)	3.00 (2.00, 4.00)	1, 5	49 (2.6%)
Q24	1864	3.71 (1.15)	4.00 (3.00, 5.00)	1, 5	30 (1.6%)
Q25	1819	4.36 (0.78)	5.00 (4.00, 5.00)	1, 5	75 (4.0%)
Q26	1862	3.44 (1.24)	4.00 (3.00, 4.00)	1, 5	32 (1.7%)
Q27	1878	3.77 (0.97)	4.00 (3.00, 4.00)	1, 5	16 (< 1%)
Q28	1885	4.25 (0.86)	4.00 (4.00, 5.00)	1, 5	9 (< 1%)
Q29	1883	3.81 (0.97)	4.00 (3.00, 5.00)	1, 5	11 (< 1%)
Q30	1840	3.46 (1.17)	4.00 (3.00, 4.00)	1, 5	54 (2.9%)
Q31	1849	4.17 (0.93)	4.00 (4.00, 5.00)	1, 5	45 (2.4%)
Q32	1846	4.19 (0.90)	4.00 (4.00, 5.00)	1, 5	48 (2.5%)
Q33	1877	4.09 (0.90)	4.00 (4.00, 5.00)	1, 5	17 (< 1%)
Q34	1879	4.69 (0.54)	5.00 (4.00, 5.00)	1, 5	15 (< 1%)
Q35	1857	4.00 (1.03)	4.00 (3.00, 5.00)	1, 5	37 (2.0%)
Q36	1821	3.62 (1.13)	4.00 (3.00, 4.00)	1, 5	73 (3.9%)
Q37	1872	4.32 (0.84)	5.00 (4.00, 5.00)	1, 5	22 (1.2%)
Q38	1797	1.88 (1.26)	1.00 (1.00, 3.00)	1, 5	97 (5.1%)
Care team domain^a^	1745	3.75 (0.84)	3.80 (3.20, 4.40)	1, 5	149 (7.9%)
What matters to me domain^a^	1509	3.83 (0.77)	3.96 (3.39, 4.39)	1, 5	385 (20.3%)
Living with Crohn's or Colitis domain^a^	1693	3.85 (0.64)	3.92 (3.50, 4.33)	1.42, 5	201 (10.6%)
Total PREM score^a^	1467	3.83 (0.70)	3.93 (3.42, 4.33)	1.24, 5	427 (22.5%)

^a^ Domain and total PREM scores were calculated only where all items in the domain/whole PREM were complete.

##### 2.7.8.3 Reliability

###### 2.7.8.3.1 Internal Consistency

Cronbach's alpha for the 45 items in the PREM was 0.97 (95% CI 0.97–0.97; *n* = 1467), demonstrating excellent internal consistency. Likewise, the ‘care team’, ‘what matters to me’ and ‘living with Crohn's or Colitis’ domains had scores of 0.85 (95% CI 0.84–0.86; *n* = 1745), 0.97 (95% CI 0.96‐0.97; *n* = 1509), and 0.88 (95% CI 0.87‐0.89; *n* = 1693) respectively, all passing the threshold for suitable internal consistency.

###### 2.7.8.3.2 Test‐Retest Reliability

The ICC indicated that the PREM has good retest reliability. For participants whose experience was ‘about the same’ on the experience anchor question between baseline and 2 weeks follow‐up (*n* = 222), the ICC was 0.88 (95% CI 0.85–0.91; *n* = 165) for the total PREM score, 0.82 (95% CI 0.77‐0.86; *n* = 205) for the ‘care team’ score, 0.87 (95% CI 0.83–0.90, *n* = 174) for the ‘what matters to me’ score and 0.84 (95% CI 0.80–0.88, *n* = 198) for the ‘living with Crohn's or Colitis’ score.

**Figure 1:**
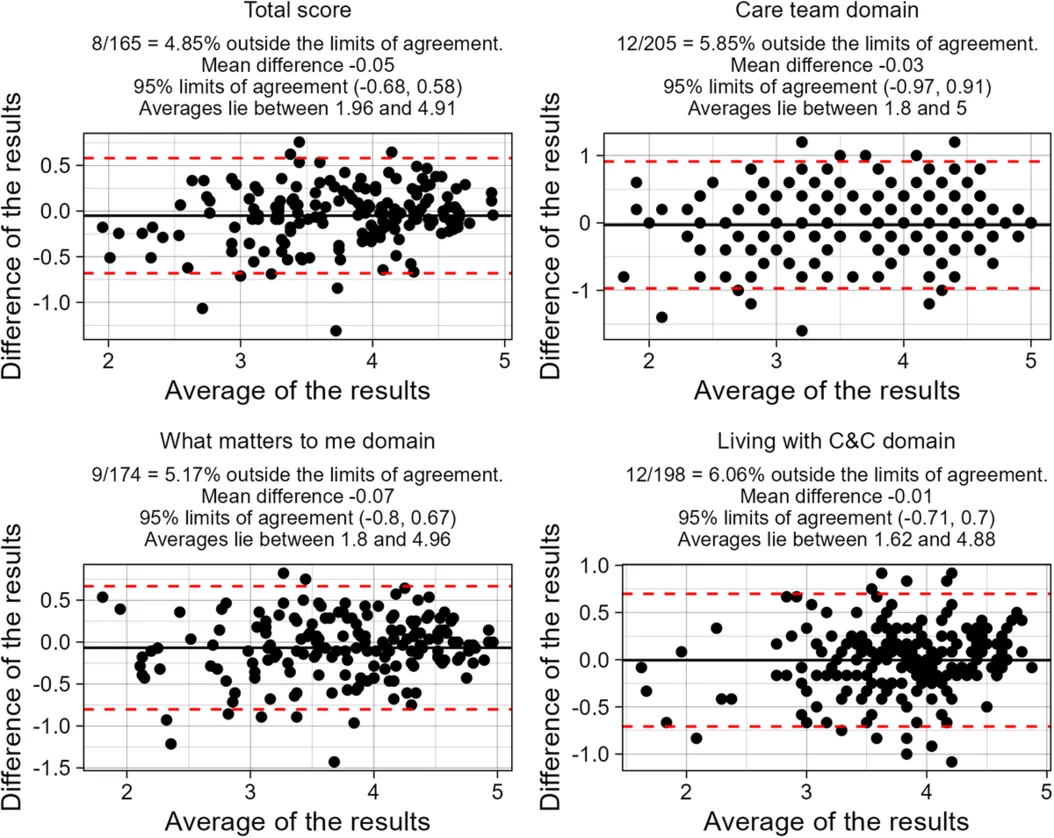
Bland–Altman plot of agreement between baseline and 2‐week follow‐up of the total PREM score and the three domain scores.

The mean difference and 95% CI between baseline and 2‐week follow‐up for these participants was −0.05 (95% CI −0.10 to −0.00, *n* = 165) for the total PREM score. When considering the separate domains, the ‘what matters to me’ domain displayed the largest difference of −0.07 (95% CI −0.12 to −0.01, *n* = 174), but the ‘care team’ and ‘living with Crohn's or Colitis’ domains suggested good retest reliability with mean differences of −0.03 (95% CI −0.10 to 0.04, *n* = 205) and −0.01 (95% CI −0.06 to 0.05, *n* = 198), respectively. No specific patterns were identified in the Bland‐Altman plots (Figure 1).

##### 2.7.8.4 Validity

###### 2.7.8.4.1 Construct Validity

Participants with active disease, as defined using the IBD‐Control‐8, reported lower scores on the ‘living with Crohn's or Colitis’ domain at baseline compared to those with non‐active disease by 0.39 (95% CI 0.22–0.55, *n* = 211). Although the value appears small, it is statistically significant (*p* < 0.001) and equates to a standardised effect size of 0.62 (95% CI 0.34–0.90, *n* = 211).

### 2.8 Structural Validity

Results of the CFA (*n* = 1467) suggested that the observed data did not support the three‐factor (domain) model. Correlations between items within the same domain ranged between 0.07 and 0.91, with the low correlations suggesting potential domain misspecification. The items in the ‘living with Crohn's or Colitis’ domain are particularly poorly correlated and item 38, about the patient's written care plan, has the lowest correlation with all items.

The chi‐square test of model fit indicated a significant difference between the factor model and the data (chi‐square = 9359, df = 942, *p* < 0.0001) but this measure is sensitive to large sample sizes. Other model fit indices also suggested poor model fit [Comparative Fit Index (CFI) 0.797; Root Mean Square Error of Approximation (RMSEA) 0.087], with the exception of the Standardised Root Mean Square Residual (SRMR) which indicated good model fit (SRMR 0.059).

There were several (42%) factor loadings below 0.7 (R‐squared < 0.5) which indicates that the chosen factors did not explain more than 50% of the variance present for those items. Several pairs of items had correlation residuals greater than |0.1| which suggested there were multiple ill‐fitted relationships in the model. Given some items had high correlations to one another and lower correlations to other items in the same domain, it would be indicative that a model with greater than three factors would be more appropriate. The modification indices proposed additional correlations, such as between items which ask about the information given by the Care Team (Q21a to Q21e).

On this basis, initial EFA was performed to generate potentially more appropriate domain solutions for further research. This indicated a four to seven factor model would be more appropriate for the data. Information relating to the assumption checking for the CFA and the EFA can be found in Appendix 3.

### 2.9 Criterion Validity

The Pearson's correlation coefficient for the total PREM score and the total IBD‐Control‐8 score across all timepoints was 0.39 (95% CI 0.34–0.44, *n* = 1117), and 0.34 (95% CI 0.29–0.39, *n* = 1230) for the IBD‐Control VAS. These are moderate correlations, as expected.

#### 2.9.1 Responsiveness

For the domain scores, there was a maximum of 5.3% of responses with either the ceiling (score of 5) or floor (score of 1) value. The total score had 0.4% (*n* = 1467) ceiling effects and no floor effects, thus there were less than 70% responses in either the highest or lowest category for domains and total score.

The correlation between the change in total PREM score and IBD‐Control score from baseline to 12 months was 0.29 (95% CI 0.09–0.46, *n* = 98). This was not expected to be perfectly correlated, however is a weak correlation.

With a SD of 0.71 (of the repeated measures), the SEM was 0.23 and subsequent smallest detectable difference was 0.64, in the unit of measurement of the PREM.

#### 2.9.2 Interpretability

The correlation between the change in total PREM score from baseline to 12 months and the experience anchor question was 0.51 (95% CI 0.37–0.63, *n* = 123), demonstrating suitable change to calculate an important difference [35]. The subset of participants that scored ‘a little bit worse’ or ‘a little bit better’ on the anchor measure at 12 months consisted of one individual whose mean absolute difference over 12 months was 0.4. With only one participant in this category, confidence intervals and the MID lower bound could not be calculated. An alternative method (distribution method) to calculate the MID used half the SD of the total PREM scores at 12 months (SD 0.726, *n* = 146), resulting in a value of 0.36.

## 3 Discussion

The IBD‐PREM demonstrated strong psychometric properties with good test–retest reliability (ICC = 0.88) and acceptable validity. High internal consistency (*α* = 0.97) was found and along with the CFA results suggests that item reduction is required and the three‐factor structure requires refinement. The instrument shows acceptability through high response rates, low missing data on most items and an average of approximately 5 min completion time.

This work has several strengths, including a large sample of 287 participants providing 1894 responses, a longitudinal design, and comprehensive psychometric testing on a measure which was created through a patient‐led development process [18]. However, some limitations are the recruitment from a single centre and a predominantly White British sample (89.2%), which limit the generalisability of the findings. While the sample captured a diverse range of socio‐economic contexts and reflected the national average, as measured by the Index of Multiple Deprivation, we acknowledge the limitation that area‐level indices do not fully capture individual lived experiences [36]. Future validations would benefit from adopting contemporary non‐deficit frameworks for reporting demographic diversity. The ability for patients to respond to the survey through a web‐based portal or by post showed that online was by far the more common response method. However, this flexibility allows options for participants that are less comfortable with online surveys.

The response rate of 68.3% at 12 months may introduce attrition bias, however this is in line with other longitudinal observational studies where 1‐year follow up ranges from 46% to 100% [35]. Furthermore, there were insufficient data to calculate an anchor‐based minimally important difference. There was no gold standard measure available for comparison due to the limited availability of patient experience measures. The use of the IBD‐Control represented the most suitable option available. Finally, the analysis using all available complete data treated the observations as independent meaning not all subjects are treated equally, however the correlations at each time point (which account for this) produce values of similar magnitude.

The psychometric properties observed may partly reflect the patient‐led development process. The eight expert service users who developed the PREM [18] ensured that items were meaningful and clearly understood by people with IBD, using language familiar to patients rather than clinical terminology. This contrasts with many instruments in our review where patient involvement was minimal or poorly reported. The think‐aloud interviews with 18 additional patients provided iterative refinement, ensuring face validity and comprehension. This extensive patient involvement throughout development may explain why the PREM achieved higher internal consistency than most comparable instruments (which ranged from 0.63 to 0.98) [37, 38] and superior test‐retest reliability compared to several existing tools (ICCs of 0.47–0.91) [15, 39]. The results found here were in line with other co‐developed PREMs [20, 22].

Low ceiling and floor effects of the domains and total score indicate that there is good responsiveness available within the measure. The instrument's ability to differentiate between patients with active and inactive disease (effect size 0.62) suggests sensitivity to clinically meaningful differences, though as highlighted by a patient, this reflects disease impact on experience rather than service quality per se. This result has further shown the relationship between having an active disease and reporting poorer experience, specifically on the questions related to living with Chron's or Colitis, which was hypothesised. The relationship is of moderate strength suggesting that although present, it is not the whole story. When considering these results, our patient representative raised further questions about the link between active disease (which is an outcome) and experience of care. He noted that bad outcomes can co‐exist with good care experience and that a patient's needs potentially change during periods of active disease. Further exploration into this complex relationship of outcomes and experience would be of interest.

Although overall completion rates remained acceptable, missing data reached 15.1% (Q18b–co‐ordination between my Care Team and other healthcare professionals). All items with missing data higher than 5% (Q3, Q18a, Q18b, Q18d, Q19, Q38) related to services or experiences that the respondents may not have had recently. Patient input has indicated that it is unlikely the respondent would know about the co‐ordination between different specialties within the hospital, hence creating the higher missing data levels which were not present in the question regarding GP and consultant co‐ordination which, our patient representative felt, is more likely to be known. Future use of the IBD‐PREM should therefore consider the ability to skip questions if they are not relevant for the respondent as a forced response may not be meaningful. The result of 22.5% of responses missing at least one question supports the potential need for item reduction.

The moderate correlation between the IBD‐PREM and IBD‐Control‐8 reflects that these instruments measure different constructs (experience and health outcomes) and IBD‐Control‐8 is not a true gold standard comparison for the PREM. Additionally, they are evaluating different timeframes with the IBD‐Control‐8 asking about the last 2 weeks and the IBD‐PREM implying a more long‐term timeframe for responses. The lack of available data for those that indicated a small change on the IBD‐PREM over 12‐month limits the ability to calculate a MID using the anchor‐based method.

The current three‐factor structure showed poor fit in CFA, suggesting the need for refinement of the conceptual model. Assessment of factor loadings indicated some items appeared to be redundant (Q25 and Q38) and others (Q26, Q27 and Q36) overlapped domains so require clarification. Discussion with our patient representative suggested that on reflection, the care team domain especially could be restructured and naturally fits into smaller groupings. Q38 (asking about the care plan) particularly is of difficulty as this appeared to be redundant and had high missing data, our patient input suggests that the wording of this could be improved to make this clearer for those patients that don't know about their care plan and to identify the difference between those that don't have one or don't know if they have one, which is an important distinction. Due to the extremely high internal consistency found, the potential for item reduction should be considered in future work. Additionally, the issues found in the CFA mean a different domain structure should be considered for future versions of the IBD‐PREM. Based on discussions with our patient representative; this could be done in conjunction with patients as with the original development of the measure.

As this work was completed in a single centre within the United Kingdom, additional validation is needed for generalisability (multi‐centre validation), diverse populations (cross‐cultural validation), younger adults (under aged 25) and vulnerable groups such as those with cognitive impairments, multimorbidity or low literacy levels [40]. This additional work should also consider any systematic missing data at the individual level. Any translations from English should consider cultural appropriateness. Despite positive findings on the validity of the PREM, additional research is needed to establish the MID and assess responsiveness to service improvements for the IBD‐PREM. Finally, in resource‐constrained settings, care prioritisation is essential; using experience data for quality improvement can be resource‐intensive [41]. Future research should therefore explore models to identify how to use data from the IBD‐PREM for maximum impact on patient experience of IBD services.

Patient‐reported data tends to show a higher value for general satisfaction compared to patient experience measures [41] which highlights the importance of the IBD‐PREM in identifying specific experiential areas rather than relying solely on satisfaction metrics. The intended use of the IBD‐PREM in service evaluations and quality improvement initiatives has been shown to be plausible due to the suitable validity and reliability found within the measure. This can guide services in identifying specific areas of care that require attention, provided there is a shared stakeholder vision, appropriate timing of feedback, and support for interpreting results [42]. This was tested within the wider AWARE‐IBD study and will be reported elsewhere to assess the implementation of the measure and assessment of its intended use in a real‐world setting.

## 4 Conclusion

The IBD‐PREM demonstrates strong reliability and validity as a measure of patient experience in inflammatory bowel disease services. With excellent internal consistency and good test‐retest reliability, it provides a robust tool for services to identify specific areas for improvement based on what matters most to patients. While structural refinement and broader validation are needed, the IBD‐PREM offers a valuable alternative to satisfaction measures by capturing the reality of care experiences. Its alignment with quality standards and ability to identify experience dimensions beyond current policy frameworks positions it as an important instrument for driving patient‐centred improvements in IBD care. Future research should investigate whether patient‐led development consistently yields superior psychometric properties compared to traditional researcher‐led approaches, as our findings suggest this methodology may be key to developing valid and reliable PREMs.

## Author Contributions

**Zoe Guy:** methodology, formal analysis, visualization, writing – original draft. **Daniel Hind:** writing – original draft, conceptualization, funding acquisition, methodology. **Nikki Totton:** conceptualization, funding acquisition, methodology, formal analysis, writing – review and editing, supervision. **Elena Sheldon:** conceptualization, funding acquisition, data curation, project administration, writing – review and editing. **Naseeb Ezaydi:** writing – review and editing, data curation, project administration. **Matt Bursnall:** methodology, supervision, writing – review and editing. **Alan Lobo:** conceptualization, investigation, funding acquisition, data curation, supervision, writing – review and editing.

## Ethics Statement

The study received ethical approval from Wales REC 3 (ref 298930). Participants gave informed consent for data collection and use in the research evaluation.

## Conflicts of Interest

The authors of this manuscript have the following competing interests: Professor Alan Lobo has acted as a consultant and advisory board member for Takeda Pharma, Janssen and Bristol Myers Squibb.

## Supporting information


Supporting File 1

Supporting File 2

Supporting File 3

## Data Availability

The data that support the findings of this study are available from the corresponding author upon reasonable request.
