# Supplementary material for: Validation of a Patient‐Created, Patient‐Reported Experience Measure for Inflammatory Bowel Disease in the United Kingdom
Source: Health Expect. 2026 Sep 9;29(5):e70814. doi: 10.1111/hex.70814 (PMC13559181; doi:10.1111/hex.70814)
Supplement: Supplementary file 1 [file HEX-29-e70814-s001.docx]

Supplementary material

[Appendix 1 - Patient-Reported Experience Measure	2](#_Toc216280642)

[Appendix 2 – Thresholds for statistical test results	5](#_Toc216280643)

[Appendix 3 – Factor Analysis	6](#_Toc216280644)

[Appendix 3.1 Assumption checking of Confirmatory Factor Analysis	6](#_Toc216280645)

[Appendix 3.2 Exploratory Factor Analysis	6](#_Toc216280646)

## Appendix 1 - Patient-Reported Experience Measure

**PLEASE READ THE STATEMENTS BELOW AND INDICATE HOW MUCH YOU *AGREE* WITH EACH ONE**

*Please answer every question, if you can, by putting a cross in the relevant box. If you think a question is not applicable to you, then you can skip the question by leaving the box blank.*

***Some of the statements below refer to your ‘Care Team’. This means the specialist IBD (Inflammatory Bowel Disease) Team at the hospital. This may be known as the Gastroenterology Team or Gastro Team, or a different name at your hospital, but ‘Care Team’ means the staff you see for your Crohn’s or Colitis care at the hospital.***

|  |  | **1**  **Not at all** | **2**  **To a little extent** | **3**  **To some extent** | **4**  **To a large extent** | **5**  **To a very large extent** |
| --- | --- | --- | --- | --- | --- | --- |
| *The Care Team* | | | | | | |
| **1** | I know who the different people are in the Care Team looking after me |  |  |  |  |  |
| **2** | I know how to contact the Care Team between appointments if I need to |  |  |  |  |  |
| **3** | I can get a response from the Care Team by the end of the next working day when experiencing a flare |  |  |  |  |  |
| **4** | I feel that the Care Team has enough time for me when I talk to them |  |  |  |  |  |
| **5** | I know the person on the Care Team who coordinates my care |  |  |  |  |  |
| *What Matters to Me* | | | | | | |
| **6** | The Care Team understands the impact my Crohn’s or Colitis has on my life |  |  |  |  |  |
| **7** | My concerns are taken seriously when I talk to the Care Team |  |  |  |  |  |
| **8** | The Care Team ask how I feel while they are treating me |  |  |  |  |  |
| **9** | I feel I can approach the Care Team to discuss any concerns about my treatment and its effects on my life |  |  |  |  |  |
| **10** | The Care Team understands what matters to me (in healthcare and beyond) |  |  |  |  |  |
| **11** | I have the confidence to express my needs and concerns with the Care Team |  |  |  |  |  |
| **12** | I feel that the Care Team do their best to give me the care I need |  |  |  |  |  |
| **13** | I am involved in decisions about my care and treatment |  |  |  |  |  |
| **14** | I feel I have a good relationship with my Care Team |  |  |  |  |  |
| **15** | I am treated with dignity and respect by the Care Team |  |  |  |  |  |
| **16** | I usually see the same person from the Care Team at each appointment (either face-to-face, telephone or online). |  |  |  |  |  |
| **17** | The Care Team offers me appointments in a format that suits me, such as face to face, by telephone or video call |  |  |  |  |  |
| **18** | There is good coordination between the different people involved in my care and treatment:  a) Within my Care Team (doctors, IBD nurse specialists, surgeons, dietitians, for example) |  |  |  |  |  |
|  | b) Between my Care Team and other teams in the hospital that I may be in contact with (rheumatology, dermatology, obstetrics, for example) |  |  |  |  |  |
|  | c) Between my Care Team and my GP Practice |  |  |  |  |  |
|  | d) Between my Care Team and other healthcare professionals |  |  |  |  |  |
| **19** | The Care Team will refer me to other services if needed (mental health services, for example) |  |  |  |  |  |
| **20** | In general, I am able to understand all the information the Care Team gives me |  |  |  |  |  |
| **21** | Thinking about the last time I was given information by the Care Team about my care and treatment:  a) It was given in a way that was easy to understand |  |  |  |  |  |
|  | b) It met my needs |  |  |  |  |  |
|  | c) It was relevant to me and my needs |  |  |  |  |  |
|  | d) I had the opportunity to discuss and ask questions about it |  |  |  |  |  |
|  | e) I liked the way it was given (e.g. verbal or on paper) |  |  |  |  |  |
| **22** | The Care Team has recommended or directed me to good, reliable information resources, such as charities and the NHS website |  |  |  |  |  |
| **23** | The Care Team informs me about opportunities to take part in research studies and clinical trials |  |  |  |  |  |
| **24** | The frequency of my routine appointments is acceptable |  |  |  |  |  |
| **25** | I am able to easily access toilet facilities at the hospital |  |  |  |  |  |
| **26** | I know how to provide feedback on the service, should I want to |  |  |  |  |  |
| *Living with Crohn’s or Colitis* | | | | | | |
| **27** | I know what care and treatment options are available for *my* Crohn’s or Colitis |  |  |  |  |  |
| **28** | I understand how Crohn’s or Colitis affects me physically |  |  |  |  |  |
| **29** | In general, I feel that I can mentally cope with my Crohn’s or Colitis |  |  |  |  |  |
| **30** | I feel able to discuss my mental health with the Care Team if I want to |  |  |  |  |  |
| **31** | I *can do* all the tasks that my care team ask me to do at home (such as manage my diet, lifestyle, treatment) |  |  |  |  |  |
| **32** | I *remember to do* all of the tasks that my care team ask me to do (such as take tablets, keep a food diary, etc.) |  |  |  |  |  |
| **33** | I am able to keep track of my symptoms |  |  |  |  |  |
| **34** | I feel it is important to take an active role in my own healthcare |  |  |  |  |  |
| **35** | I get enough support from the people around me to help me live with Crohn’s or Colitis (such as friends, family or people at work) |  |  |  |  |  |
| **36** | I can access support from the IBD community to help me live with Crohn’s or Colitis, if I want to (such as charities, online groups, support groups) |  |  |  |  |  |
| **37** | I believe that my care and treatment will benefit me |  |  |  |  |  |
| **38** | I have a personalised written care plan* |  |  |  |  |  |

**Personalised written care plan definition: This is different to a first treatment plan. A care plan is a document that sets out how your Crohn's or Colitis will be treated, managed and monitored. It should take into consideration your hopes and life aspirations. It is an agreement or plan between you and your healthcare professional to help meet those goals. This is different to the letters or correspondence that a doctor may send you or your GP after each appointment.*

**Thank you for taking the time to complete this survey.**

## Appendix 2 – Thresholds for statistical test results

Table 4: Acceptable limits as defined in the literature for different statistical tests ^52^

| **Assessment** | **Acceptable Limit** |
| --- | --- |
| Cronbach’s Alpha | ≥0.70 |
| Pearson’s Correlation Coefficient | ≥0.70  ≥0.60 for construct validity (4)  ≥0.4 for responsiveness |
| Intraclass Correlation Coefficient | ≥0.70 |
| Ceiling/Floor | <70% in highest or lowest category |
| Effect Sizes | 0.2 - 0.5 = small,  0.5 - 0.8 = moderate,  0.8 + large (7)  ≥0.2 for smallest detectable difference |
| Model fit for confirmatory factor analysis | CFI >0.95  SRMR <0.08  RMSEA <0.06 |

## Appendix 3 – Factor Analysis

### Appendix 3.1 Assumption checking of Confirmatory Factor Analysis

Complete cases were used in the CFA (n=1467) due to low levels of missing data and missingness indicated to be random (Little’s MCAR test: chi-square = 1823.04, df = 9817, p = 1.000). Average item scores ranged from 1.98 to 4.70. Six responses (6/1467, 0.4%) had maximum total PREM scores (score of 5) and no responses had minimum total PREM scores (score of 1). Majority of items had a skewness and kurtosis below |1| (64% and 67% respectively), all items had a skewness below |2| and kurtosis below |4|. The data showed significant multivariate skewness and kurtosis (Mardia’s multivariate normality test, p-values <0.0001, implemented by the psych package). The correlations between items within the same domain ranged between 0.07 and 0.91.

### Appendix 3.2 Exploratory Factor Analysis

Confirmatory factor analysis (CFA) suggested that the data may be better represented by more than three domains. Exploratory factor analysis (EFA) was performed to explore possible other solutions.

Parallel analysis of principal components and factor analysis (based on eigenvalues) indicated 3 components and 8 factors. Considering solutions with eigenvalues greater than 1 would suggest between 3 and 7 factors. Levelling out occurred between 4 and 5 and between 8 and 9 factors. Therefore, models with between 4 and 8 factors were run and the factor loadings of the four-factor solution were presented. There were patterns occurring across the various models. Items originally in the ‘care team’ domain were consistently split between two factors. Items that were in the ‘living with Crohn’s or Colitis’ domain tended to remain within the same factor, with the exception of q30 and q38. The items in the ‘what matters to me’ would then be divided among the remaining factors in the model in similar clusters of items (e.g. q6-q15). There were several items with consistently low factor loadings across models and would be potential candidates for removal from the questionnaire. Communality and complexity measures suggested q25 and q38 were poorly explained by all models, and q25, q26, q27 and q36 typically required multiple factors to explain them. In total, 56% of the variance in the data was explained by the four-factor model and 61% of the variance in the data was explained by the seven-factor model. All the models presented explain greater than 50% of the variance in the data, but given there were a number of poorly fitted items in each model, it would be recommended to first remove some items and then perform another round of EFA prior to confirming a suitable model.

Table 5: Standardised pattern coefficients for the PREM for the four-factor solution. Bold typeface indicates the proposed factor for the item, italic typeface indicates a poorly fitted item to the model.

| **Item** | **PA1** | **PA2** | **PA3** | **PA4** | **Communality** | **Uniqueness** | **Complexity** |
| --- | --- | --- | --- | --- | --- | --- | --- |
| q1 | 0.21 | 0.03 | **0.52** | -0.09 | 0.47 | 0.53 | 1.4 |
| q2 | **0.50** | 0.01 | 0.29 | -0.17 | 0.52 | 0.48 | 1.9 |
| q3 | 0.46 | -0.09 | 0.36 | -0.16 | 0.48 | 0.52 | 2.3 |
| q4 | **0.68** | -0.03 | 0.17 | -0.07 | 0.61 | 0.39 | 1.1 |
| q5 | 0.18 | 0.00 | **0.54** | -0.04 | 0.46 | 0.54 | 1.2 |
| q6 | **0.65** | -0.03 | 0.10 | 0.27 | 0.62 | 0.38 | 1.4 |
| q7 | **0.88** | -0.06 | -0.05 | 0.15 | 0.72 | 0.28 | 1.1 |
| q8 | **0.69** | 0.01 | 0.02 | 0.23 | 0.61 | 0.39 | 1.2 |
| q9 | **0.78** | -0.01 | 0.07 | 0.19 | 0.76 | 0.24 | 1.1 |
| q10 | 0.54 | -0.02 | 0.26 | 0.33 | 0.72 | 0.28 | 2.1 |
| q11 | **0.73** | 0.08 | -0.01 | 0.23 | 0.70 | 0.30 | 1.2 |
| q12 | **0.86** | -0.05 | 0.05 | 0.04 | 0.75 | 0.25 | 1.0 |
| q13 | **0.54** | 0.13 | 0.16 | 0.04 | 0.57 | 0.43 | 1.3 |
| q14 | **0.66** | -0.02 | 0.27 | -0.02 | 0.74 | 0.26 | 1.3 |
| q15 | **0.77** | -0.02 | 0.01 | -0.01 | 0.58 | 0.42 | 1.0 |
| q16 | 0.14 | 0.04 | **0.53** | -0.07 | 0.42 | 0.58 | 1.2 |
| q17 | *0.38* | *0.13* | *0.26* | *-0.04* | 0.45 | 0.55 | 2.1 |
| q18a | 0.36 | 0.01 | **0.54** | -0.05 | 0.69 | 0.31 | 1.8 |
| q18b | -0.06 | 0.01 | **0.81** | -0.03 | 0.59 | 0.41 | 1.0 |
| q18c | 0.19 | 0.03 | **0.55** | -0.04 | 0.49 | 0.51 | 1.3 |
| q18d | -0.06 | 0.05 | **0.84** | 0.03 | 0.68 | 0.32 | 1.0 |
| q19 | 0.05 | 0.15 | **0.63** | 0.17 | 0.62 | 0.38 | 1.3 |
| q20 | 0.54 | 0.33 | 0.07 | -0.08 | 0.69 | 0.31 | 1.7 |
| q21a | **0.76** | 0.16 | -0.02 | -0.17 | 0.72 | 0.28 | 1.2 |
| q21b | **0.81** | 0.12 | -0.01 | -0.17 | 0.77 | 0.23 | 1.1 |
| q21c | **0.79** | 0.15 | -0.01 | -0.16 | 0.77 | 0.23 | 1.2 |
| q21d | **0.75** | 0.11 | 0.00 | -0.08 | 0.66 | 0.34 | 1.1 |
| q21e | **0.70** | 0.16 | 0.05 | -0.14 | 0.69 | 0.31 | 1.2 |
| q22 | *0.20* | *0.13* | *0.37* | *0.13* | 0.41 | 0.59 | 2.1 |
| q23 | *0.16* | *0.19* | *0.27* | *0.16* | 0.32 | 0.68 | *3.3* |
| q24 | **0.45** | 0.15 | 0.20 | 0.00 | 0.50 | 0.50 | 1.6 |
| q25 | *0.10* | *0.23* | *0.20* | *0.05* | *0.21* | 0.79 | 2.5 |
| q26 | *0.19* | *0.22* | *0.31* | *0.20* | 0.46 | 0.54 | *3.4* |
| q27 | 0.29 | **0.41** | 0.18 | 0.09 | 0.59 | 0.41 | 2.4 |
| q28 | -0.01 | **0.55** | 0.11 | 0.07 | 0.38 | 0.62 | 1.1 |
| q29 | 0.16 | **0.57** | -0.07 | 0.18 | 0.45 | 0.55 | 1.4 |
| q30 | *0.32* | *0.32* | *0.15* | *0.37* | 0.66 | 0.34 | *3.3* |
| q31 | 0.10 | **0.73** | -0.01 | -0.05 | 0.62 | 0.38 | 1.0 |
| q32 | -0.01 | **0.76** | -0.01 | -0.08 | 0.57 | 0.43 | 1.0 |
| q33 | -0.07 | **0.73** | 0.09 | -0.04 | 0.54 | 0.46 | 1.1 |
| q34 | 0.10 | **0.52** | -0.08 | -0.06 | 0.31 | 0.69 | 1.2 |
| q35 | -0.03 | **0.54** | 0.09 | 0.18 | 0.37 | 0.63 | 1.3 |
| q36 | -0.03 | 0.44 | 0.34 | 0.15 | 0.46 | 0.54 | 2.2 |
| q37 | 0.41 | 0.44 | 0.01 | -0.02 | 0.57 | 0.43 | 2.0 |
| q38 | *-0.09* | *0.07* | *0.39* | *0.14* | *0.17* | 0.83 | 1.5 |
